# Resistance Levels and Epidemiology of Non-Fermenting Gram-Negative Bacteria in Urinary Tract Infections of Inpatients and Outpatients (RENFUTI): A 10-Year Epidemiological Snapshot

**DOI:** 10.3390/antibiotics8030143

**Published:** 2019-09-09

**Authors:** Márió Gajdács, Katalin Burián, Gabriella Terhes

**Affiliations:** 1 Department of Pharmacodynamics and Biopharmacy, Faculty of Pharmacy, University of Szeged, Eötvös utca 6, 6720 Szeged, Hungary; 2 Department of Medical Microbiology and Immunobiology, Faculty of Medicine, University of Szeged, Dóm tér 10, 6720 Szeged, Hungary; 3 Institute of Clinical Microbiology, Faculty of Medicine, University of Szeged, Semmelweis utca 6, 6725 Szeged, Hungary

**Keywords:** urinary tract infection, UTI, antibiotic, resistance, epidemiology, non-fermenting, *Acinetobacter*, *Pseudomonas*, *Stenotrophomonas*

## Abstract

*Background:* Urinary tract infections (UTIs) are one of the most common infections in the human medicine, both among outpatients and inpatients. There is an increasing appreciation for the pathogenic role of non-fermenting Gram-negative bacteria (NFGNBs) in UTIs, particularly in the presence of underlying illnesses. *Methods:* The study was carried out using data regarding a 10-year period (2008–2017). The antimicrobial susceptibility testing was performed using the disk diffusion method, E-tests, and broth microdilution. *Results:* NFGNB represented 3.46% ± 0.93% for the outpatients, while 6.43% ± 0.81% of all positive urine samples for the inpatients (*p* < 0.001). In both groups, *Pseudomonas* spp. (78.7% compared to 85.1%) and *Acinetobacter* spp. (19.6% compared to 10.9%), were the most prevalent. The *Acinetobacter* resistance levels were significantly higher in inpatients isolates (*p* values ranging between 0.046 and <0.001), while the differences in the resistance levels of *Pseudomonas* was not as pronounced. The *β*-lactam-resistance levels were between 15–25% and 12–28% for the *Acinetobacter* and *Pseudomonas* spp., respectively. 4.71% of *Acinetobacter* and 1.67% of *Pseudomonas* were extensively drug resistant (XDR); no colistin-resistant isolates were recovered. *Conclusions:* Increasing resistance levels of the *Acinetobacter* spp. from 2013 onward, but not in the case of the *Pseudomonas* spp. Although rare, the drug resistant NFGNB in UTIs present a concerning therapeutic challenge to clinicians with few therapeutic options left.

## 1. Introduction

Urinary tract infections (UTIs) are the second most common type of infections in the human medicine in the United States and Europe and the third most common (following respiratory tract infections and gastrointestinal infections) infectious pathologies worldwide, representing an important factor of morbidity and mortality, both among outpatients and hospitalized patients (in the latter group, they may represent 25–50% of infections overall) [1,2,3]. UTIs are a considerable economic burden for healthcare institutions and national economies; additionally, they also have a substantial economic impact, as they result in lost working days [4,5]. In fact, the annual cost of UTIs in the US has been estimated to be more than 3.5 billion US dollars [6]. The principal causes of uncomplicated and community-associated UTIs are the members of the *Enterobacteriaceae* family (or more recently, the *Enterobacterales* order), namely the *Escherichia coli* and *Klebsiella* spp. in the highest numbers, while the CES [*Citrobacter*-*Enterobacter*-*Serratia*] group and members of the *Proteae* tribe are represented in the lesser numbers [1,2,3,7,8,9,10,11,12,13]; nevertheless, the etiological spectrum of nosocomial infections is more diverse, with non-fermenters, *Staphylococcus aureus*, *S. saphrophyticus*, *Enterococcus* spp., and *Candida* spp. in the higher numbers [2,14,15,16,17,18].

Non-fermenting Gram-negative bacteria (NFGNB) are a heterogenous group of *Proteobacteria*, which are characterized by the inability to ferment sugars to generate energy for their vital cellular functions. NFGNB include (in a decreasing order of prevalence) *Pseudomonas*, *Acinetobacter*, the *Burkholderia cepacia complex* (BCC), *Stenotrophomonas* (*Xanthomonas*) *maltophilia*, in addition to some less frequently isolated genera, such as *Achromobacter*, *Alcaligenes*, *Brevimundas*, *Elisabethkingia*, *Flavobacterium* and *Ralstonia* among others [19,20]. Some less prevalent members, such as *B. mallei* and *B. pseudomallei* even possess the relevance as bioterrorism agents [21,22]. These microorganisms are ubiquitous in nature, especially in aquatic environments and on abiotic surfaces, in addition to being associated with plants pathologies [19,23]. In humans, they are most frequently isolated from respiratory tract samples (they are especially important in cystic fibrosis patients) and from invasive infections (bacteremia, sepsis), however, the pathogenic role of these microorganisms has been described in a variety of other clinical situations [24,25,26]. They are extremely prevalent in opportunistic infections, affecting severely immunocompromised, debilitated patients over 60 years of age [19,20,24,26,27,28]. Interpretation of the NFGNB-positivity may be tricky for clinical microbiologists, as their true significance (contaminant, colonizer or true pathogen) should be ascertained based on the patient’s symptoms and the presence of relevant risk factors [29,30]. The introduction of the matrix-assisted laser desorption/ionization time-of-flight mass spectrometry (MALDI-TOF MS) in routine laboratories has revolutionized clinical microbiology diagnostics [31,32]. The use of this technology (based on measuring the spectra of conserved, ribosomal proteins of relevant microorganisms) has brought forward a significant change in the detection of NFGNB as well, allowing for the timely and precise identification of several species, which previously only could be differentiated by the use of molecular methods (e.g., polymerase chain reaction) [33,34,35].

There is an increasing appreciation for the pathogenic role of NFGNB in urinary tract infections, particularly in children and in patients, who are present with underlying factors that predispose them to the development of complicated UTIs, e.g., developmental abnormalities, obstruction, vesicourethral reflux, Type II diabetes, immunosuppression (corresponding to diseases or iatrogenic factors, such as the therapeutic use of corticosteroids, mycophenolate mofetil or methotrexate), cancer or others [36,37,38]. Additionally, the urinary catheterization is one of the most important factors, predisposing patients to the development of UTIs [2,18]. The NFGNB possess lipopolysaccharide (LPS), various adhesins, flagella, pili, and they are characterized by the production of biofilms, cytotoxins (exotoxin A, exoenzyme S), and toxic pigments (pyoverdine, pyocyanin, pyomelanine), proteases, hemolysin, and siderophores; all these virulence factors may have a role in the pathogenesis of UTIs, especially if the infection occurs through the intraluminal (catheter-associated) route [36,39,40,41,42,43]. In addition, there is extensive literature regarding the proclivity of NFGNB as multidrug resistant (MDR) pathogens. The therapy of MDR UTIs is a serious concern for clinicians, as there are few therapeutic options available, especially if some agents are further excluded due to intrinsic resistance mechanisms [36,38,39,40,44,45]. The etiological spectrum and the prevalence of individual pathogens in UTIs may vary significantly in different geographical regions or healthcare settings. In addition, treating physicians, armed with the knowledge of regional epidemiological (prevalence) and non-susceptibility levels, may choose the appropriate antimicrobial therapy for their patients more effectively [46,47]. The aim of this study was to report the prevalence and the temporal changes in the susceptibility levels of NFGNB in the urinary tract infections of inpatients and outpatients, using the methods of analytical epidemiology at a tertiary-care center in Hungary retrospectively, during a 10-year study period (2008–2017).

## 2. Results

### 2.1. Demographic Characteristics, Sample Types

**Figure 1:**
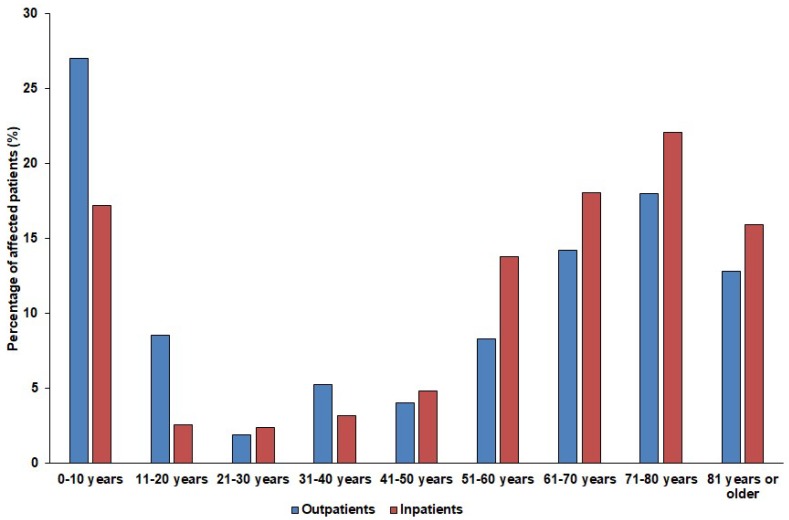
Age distribution of the affected patients in the outpatient and inpatient group.

The median age of affected patients was 67 years (range: 0.2–99; median_2008-2012_: 64 years; range: 0–95; median_2013-2017_: 69; range: 0–99; *p* > 0.05) in the outpatient group with a female-to-male ratio of 0.48 (32.3% female), while in the inpatient group, these values were 56 years (range: 0.7–95 years; median_2008-2012_: 45 years; range: 0–88; median_2013-2017_: 62 years; range: 0–95; *p* = 0.032) and 0.59 (37.2% female), respectively. The detailed age distribution of the patients in both affected patient groups is presented in Figure 1. The difference in the age distribution of the inpatient and outpatient groups was statistically significant (*p* = 0.0013). Among the affected patients, the age groups of 10 years or younger (outpatients: 27.0%, inpatients: 17.2%) and patients over 60 years of age (outpatients: 44.9%, inpatients: 56.1%) were the most numerous.

During the 10-year study period, the Institute of Clinical Microbiology received 21,150 urine samples from outpatient clinics and 19,325 samples from inpatient departments that turned out to be positive for a significant urinary pathogen. All samples (100%; *n* = 731) received from outpatient clinics were voided (midstream) urine, while the sample distribution from the inpatient departments was the following: Catheter-specimen urine (72.4%), midstream urine (24.4%), first-stream urine (2.3%), and samples obtained through a suprapubic bladder aspiration (0.8%).

### 2.2. Distribution of Non-fermenting Gram-negative Bacteria in Urine Samples

**Figure 2:**
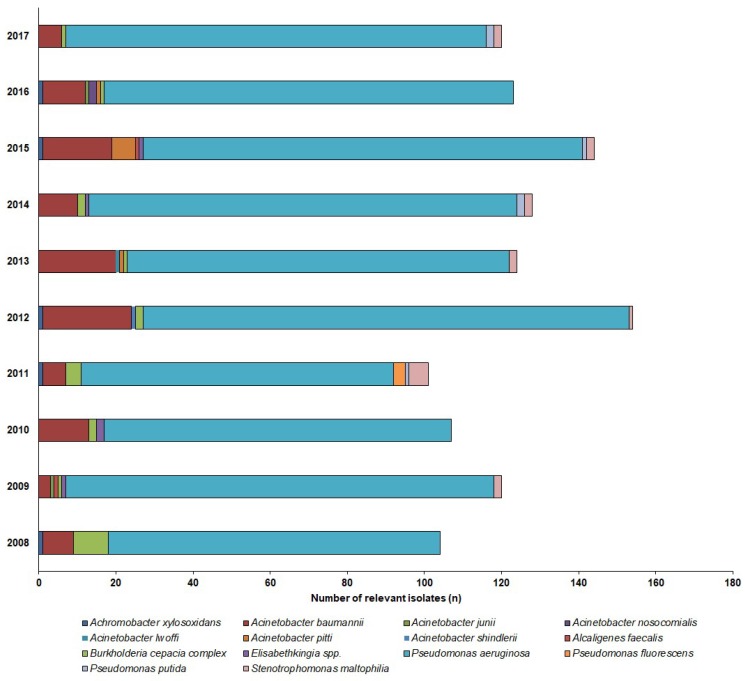
Frequency and species distribution of non-fermenting Gram-negative bacterial (NFGNB) isolates in the outpatient samples (2008—2017).

**Figure 3:**
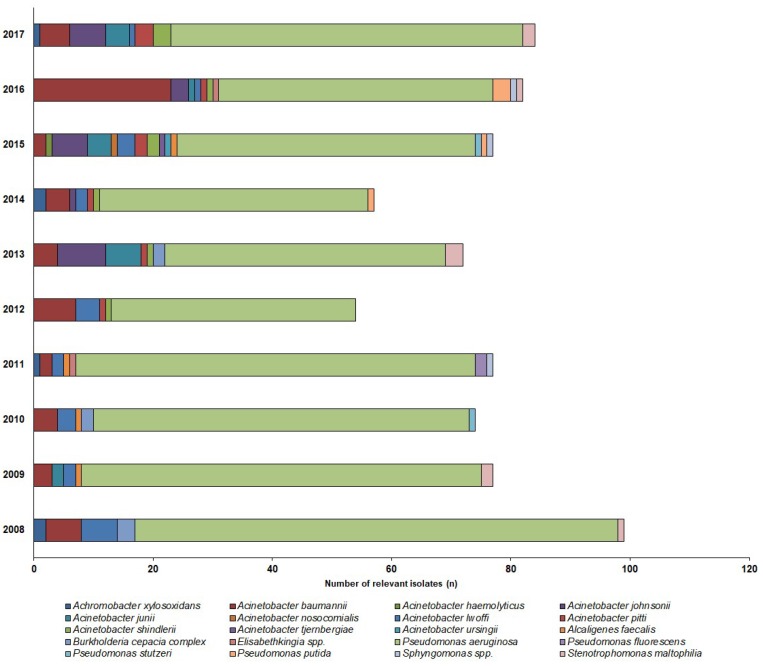
Frequency and species distribution of non-fermenting Gram-negative bacterial (NFGNB) isolates in the inpatient samples (2008—2017).

731 NFGNB isolates were obtained from outpatients (73.1 ± 11.9/year; range: 54–99) and 1229 from inpatients (122.9 ± 15.6/year; range: 104–144), corresponding to *n* = 649 outpatients and *n* = 1084 inpatients. Thus, out of the positive urine samples, these pathogens represented 3.46% ± 0.93% (range: 2.52–5.53%) for outpatients, while 6.43% ± 0.81% (range: 5.61–7.84%) of all positive urine samples for inpatients; (*p* < 0.001). In both groups, the *Pseudomonas* spp. (outpatients: 78.7%; inpatients: 85.1%; mainly *P. aeruginosa*, >99%) and *Acinetobacter* spp. (outpatients: 19.6%, inpatients: 10.9%), were the most prevalent, while the other species, e.g., *S. maltophilia*, *Alcaligenes* spp., *B. cepacia* complex, *Elisabethkingia* spp., *Sphynogomonas* spp.) were in a minority (inpatients: 4%, outpatients: 1.9%). The epidemiology and detailed species distribution of the samples in both patient groups are presented in Figure 2 (inpatients) and Figure 3 (outpatients). In the inpatient group, 14 different species of NFGNB were isolated (median: 6; range: 4–8), while in the outpatient group, the species distribution was more diverse, with 20 different species (median: 11; range: 4–15) detected.

### 2.3. Antibiotic Resistance Levels Among Urinary Non-fermenting Gram-negative Bacteria

The resistance levels of *Acinetobacter* and *Pseudomonas* isolates against the relevant antibiotics are presented in Table 1 and Table 2, respectively. To identify temporal developments in the resistance levels, the 10-year study period was divided into two five-year periods (2008–2012 and 2013–2017, respectively). The level of resistance in the *Acinetobacter* species was significantly higher in (*p* values ranging between 0.046 and <0.001) in the isolates originating from inpatients, in both study periods (excluding SMX/TMP resistance in the second half of the study period), the ratio of resistant isolates was 3–10-times higher between 2008–2012, while 3–5-times higher during 2013–2017. The differences in the resistance levels of *Pseudomonas* spp. was not as pronounced: While in the first part of the study period, there was a significant difference among the inpatient/outpatient isolates (*p* values ranging between 0.033–0.045; excluding amikacin resistance), this difference was shown only for gentamicin (*p* = 0.043), imipenem (*p* = 0.036), and meropenem (*p* = 0.029) in the second half of the study period; the ratio of the resistant isolates was 1.2–1.4-times higher between 2008–2012, while 0.8–2.2-times higher during 2013–2017. A significant increase in the resistance levels of aminoglycosides, fluoroquinolones, and SMX/TMP was demonstrated for the *Acinetobacter* spp. between 2008–2012 and 2013–2017, while similar trends were identified for imipenem, meropenem, and ceftazidime, in the case of the *Pseudomonas* spp. (*p* < 0.01). Based on the susceptibility-patterns of the individual isolates, 9.66% of the *Acinetobacter* spp. and 8.54% of the *Pseudomonas* spp. were multidrug resistant (MDR), while 4.71% of the *Acinetobacter* spp. and 1.67% of the *Pseudomonas* spp. were extensively drug resistant (XDR), during the 10-year period overall. No colistin-resistant *Acinetobacter* or *Pseudomonas* isolates were recovered from the urinary isolates during the study period. Resistance trends of the urinary *S. maltophilia* were the following: Among eight outpatient isolates, six were susceptible to SMX/TMP, five to levofloxacin, four to colistin and two to amikacin in the inpatient group, among 16 isolates, 12 were susceptible to SMX/TMP, 10 to levofloxacin, eight to amikacin and seven to colistin. 

## 3. Discussion

Non-fermenting Gram-negative bacteria present a concerning therapeutic challenge to clinicians, due to their increasing levels of resistance to several classes of antibiotics, ultimately leading to MDR, XDR or even pandrug-resistant (PDR) isolates, leading to prolonged therapy, sequelae, and excess mortality in the affected patient population [36,38,39,40,41,43,44,45,48,49,50,51,52,53]. While the most worrisome reports in the international literature have emerged regarding drug resistant *A. baumannii*, due to its much higher prevalence, the relevance of *P. aeruginosa* is not negligible, as this microorganism also has the proclivity of becoming multiple drug resistant [36,41,44,49]. Resistance in these pathogens may arise due to intrinsic non-susceptibility mechanisms, they may be acquired (mutations or through plasmids/integrons) or they may develop during prolonged therapy, which was initially effective [36,38,39,40,41,43,44,45,48,49]. The mechanism of resistance include porin loss and mutations affecting outer membrane permeability (*β*-lactam antibiotics), alterations in target sites (aminoglycosides, fluoroquinolones), energy-dependent efflux pumps (fluoroquinolones), in addition to the production of drug-inactivating enzymes (e.g., AmpC-*β*-lactamases, carbapenemases) [36,38,39,40,41,43,44,45,48,49,54,55]. In some cases, these resistance mechanisms affect the susceptibility of individual antibiotics differently (even in the same group); this is the reason why some isolates may be resistant to meropenem, but not imipenem, or resistant to amikacin, but not tobramycin. *S. maltophilia* infections are also a serious concern, as this microorganism is intrinsically resistant to a wide range of antimicrobial drugs, and data on clinical effectiveness is only available for sulfamethoxazole/trimethoprim and fluoroquinolones [56,57,58,59].

The epidemiological characteristics of this region, regarding other Gram-negative urinary pathogens has already been described previously: *E. coli* was the most prevalent (~57% for outpatients and ~42% for inpatients), followed by *Klebsiella* spp. (~8% compared to ~13%) [60], *Proteus-Morganella-Providencia* species (~5% compared to ~7%) [61], and the CES group [*Citrobacter-Enterobacter-Serratia* species] (~3% compared to ~3%) [62]. Thus, it can be concluded that NFGNB in the UTIs should not be neglected as important pathogens from an epidemiological standpoint, as their recorded prevalence was higher than of microorganisms in the CES group, and it was on par with members of the *Proteae* tribe [39]. Interestingly, the abovementioned group of bacteria are often grouped together by clinicians as “SPACE” pathogens (*Serratia, Sseudomonas, Scinetobacter, Sitrobacter and Snterobacter* spp.), as all of these bacteria possess AmpC-type *β*-lactamases in their chromosomes [63,64]. In our present study, there was a marked increase detected in the resistance levels of the *Acinetobacter* spp. in the second half of the study period (from 2013 onward), while this trend was not as pronounced in the case of the *Pseudomonas* spp., the *β*-lactam-resistance levels were between 15–25% among the *Acinetobacter* species, while for the *Pseudomonas* spp., the *β*-lactam-resistance levels were 12–28% and the aminoglycoside resistance was 13–25%. The increase in the ratio of resistant NFGNB isolates severely limits the therapeutic options available for clinicians in the infections, which is especially true for vulnerable patient populations (e.g., neonates, children, pregnant women) as some of the possible alternative drugs (fluoroquinolones, aminoglycosides) are contraindicated due to their debilitating side effects or teratogenicity [36,41,44,49,65]. In some cases, physicians have no choice but to use agents with pronounced toxicities (e.g., colistin), or newer agents with significantly higher prices (e.g., ceftazidime-avibactam, delafloxacin) [66,67]. The introduction of such novel antimicrobial drugs in the last decade may temporarily prevent the situation of untreatable infections, however, it is unknown when will they become a part of mainstream therapeutic protocols, due to financial considerations [66,68]. In addition to underlying patient factors and drug hypersensitivity, national/institutional drug availability and the local resistance profile of urinary pathogens should influence the choice of antibiotic therapy [69,70,71].

The purpose of the present study was to report on the importance of non-fermenting Gram-negative bacteria in urinary tract infections at the southern region of Hungary over a long surveillance period (10 years), in a clear and concise fashion. To the best of our knowledge, this is the longest-spanning and most detailed study originating from Hungary. The data in this study may aid the creation of a national surveillance system for urinary tract pathogens and to ascertain the relevance of non-fermenters as important uropathogens. Some limitations of this study should be noted: The retrospective design and the inability to access the medical records of the individual patients affected by these infections hindered the authors from assessing the correlation of the relevant risk factors and underlying pathologies with the NFGNB UTIs. The selection bias is a characteristic of such epidemiological studies, as most of these reports are originated from tertiary-care centers, corresponding to patients with more severe conditions or underlying illnesses [72]. Lastly, the molecular characterization of resistance determinants in the mentioned isolates was not performed, non-susceptibility was characterized by phenotypic methods only.

## 4. Materials and Methods

### 4.1. Study Location and Design, Data Collection

The present retrospective microbiological study was carried out using data collected, corresponding to the time period between 1 January 2008–31 December 2017, at the Institute of Clinical Microbiology, University of Szeged. This clinical microbiology laboratory serves the Albert Szent-Györgyi Clinical Center, which is an 1820-bed primary- and tertiary-care teaching hospital in the Southern Great Plain of Hungary (population: 401,500 people; 2017) [73]. Data collection was performed electronically, in the records of the laboratory information system (LIS), corresponding to urine samples positive for the NFGNB, based on the criteria below.

Samples with clinically significant colony counts for NFGNB (>10^5^ CFU/mL; however, this was subject to interpretation by the senior clinical microbiologists, based on the information provided on the clinical request forms for the microbiological analysis and international guidelines) that were positive for the nitrite and leukocyte-esterase tests were included in the data analysis. Only the first isolate per patient was included in the study; however, isolates with different antibiotic-susceptibility patterns from the same patient were considered as different individual isolates. To evaluate the demographic characteristics of these infections, patient data was also collected, which was limited to sex, age at the sample submission, and inpatient/outpatient status. The study was deemed exempt from ethics review by the Institutional Review Board, and informed consent was not required as data anonymity was maintained.

### 4.2. Identification of Isolates

Ten microliters of each uncentrifuged urine sample was cultured on UriSelect chromogenic agar (Bio-Rad, Berkeley, CA, USA) and blood agar (bioMérieux, Marcy-l’Étoile, Lyon, France) plates with a calibrated loop, according to the manufacturer’s instructions, and incubated at 37 °C for 24–48 h, aerobically. In the period between 2008–2012, presumptive, biochemical reaction-based methods and VITEK 2 Compact ID/AST (bioMérieux, Marcy-l’Étoile, France) were used for bacterial identification; from 2013 onward, the MALDI-TOF MS (Bruker Daltonik Gmbh., Billerica, MA., USA) was introduced to the workflow of the Department of Bacteriology. Mass spectrometry was performed by the Microflex MALDI Biotyper (Bruker Daltonics, Germany) instrument, using the MALDI Biotyper RTC 3.1 software (Bruker Daltonics, Germany) and the MALDI Biotyper Library 3.1 for the spectrum analysis. The sample preparation, methodology, and the technical details of the MALDI-TOF MS measurements were described elsewhere [74].

### 4.3. Susceptibility Testing of Relevant Isolates

Antimicrobial susceptibility testing for the *Pseudomonas* and *Acinetobacter* species was performed using the Kirby-Bauer disk diffusion method and E-tests (Liofilchem, Abruzzo, Italy) on the Mueller-Hinton agar (MHA) plates in the case of piperacillin-tazobactam, ceftazidime, cefepime, imipenem, meropenem, ciprofloxacin, levofloxacin, gentamicin, tobramycin, amikacin, and sulfamethoxazole-trimethoprim (SMX/TMP), taking into account the intrinsic resistance mechanisms of the NFGNB and the local antibiotic utilization data [44,75]. In addition, for the verification of discrepant results, the VITEK 2 Compact ID/AST (bioMérieux, Marcy-l’Étoile, France) was also utilized. Colistin susceptibility was performed using the broth microdilution method in a cation-adjusted Mueller-Hinton broth (MERLIN Diagnostik). Colistin susceptibility testing was not routinely performed, only per request of the clinicians. Susceptibility testing for the *S. maltophilia* was performed for sulfamethoxazole-trimethoprim, levofloxacin, colistin, amikacin, and tigecycline, according to a protocol previously described [57]. The interpretation of the results was based on EUCAST breakpoints (http://www.eucast.org). The *S. aureus* ATCC 29213, *E. faecalis* ATCC 29212, *Proteus mirabilis* ATCC 35659, *E. coli* ATCC 25922, *P. aeruginosa* ATCC 27853, *A. baumannii* ATCC 19606, and *S. maltophilia* ATCC 13637 were used as quality control strains. Intermediate results were grouped with and reported as resistant. Classification of the isolates as a multidrug resistant (MDR) or extensively drug resistant (XDR) was based on the EUCAST Expert Rules [76].

### 4.4. Statistical Analyses

Statistical analyses, including the descriptive analysis (means or medians with ranges and percentages to characterize data) and statistical tests (Student’s t-test [for data on resistance levels] and Mann-Whitney U test [for epidemiological data]) were performed with the SPSS software version 24 (IBM SPSS Statistics for Windows 24.0, IBM Corp., Armonk, NY, USA,). The normality of variables was tested using Shapiro–Wilk tests [for epidemiological and resistance data]. *p* values <0.05 were considered statistically significant.

## 5. Conclusions

Urinary tract infections are principally caused by members of the *Enterobacterales* (*E. coli*, *Klebsiella* spp., CES species and *Proteae*), non-fermenting Gram-negative bacteria are emerging as important causative agents of UTIs, primarily affecting elderly, hospitalized patients (characterized by co-morbidities, catheterization), both in high- and low-income countries. The emergence of drug resistance in these pathogens should be closely monitored, due to their proclivity to becoming MDR and their plasticity in drug resistance mechanisms. The present report aims to summarize the results of a long-term surveillance study of resistance levels in NFGNB originating from urine samples. Although the levels of extensively drug resistant isolates was relatively low in the southern region of Hungary (<5%), an increase in the levels of non-susceptibility to the respective antibiotics (especially in case of *Acinetobacter* spp.) was shown. For public health purposes, the continuous surveillance of resistance trends in these pathogens (both in urinary tract infections and from invasive samples) is of utmost importance.

## Figures and Tables

**Table 1:** Percentage of resistant *Acinetobacter* strains to relevant antibiotics from the inpatient and outpatient departments (2008–2017).

Tested Antibiotics	2008–2012			2013–2017		
	Outpatients	Inpatients	Statistics ^a^	Outpatients	Inpatients	Statistics ^a^
Amikacin	5.7% (*n* = 4)	57.0% (*n* = 39)	*p* < 0.001	10.95% (*n* = 8)	51.0% (*n* = 34)	*p* < 0.001
Gentamicin	7.1% (*n* = 6)	59.3% (*n* = 40)	*p* < 0.001	10.95% (*n* = 8)	41.6% (*n* = 28)	*p* < 0.001
Tobramycin	6.3% (*n* = 5)	37.4% (*n* = 25)	*p* = 0.022	10.95% (*n* = 8)	26.9% (*n* = 19)	*p* = 0.036
Ciprofloxacin	10.0% (*n* = 8)	61.1% (*n* = 41)	*p* < 0.001	17.8% (*n* = 12)	43.7% (*n* = 29)	*p* < 0.001
Levofloxacin	7.1% (*n* = 6)	53.7% (*n* = 36)	*p* < 0.001	16.4% (*n* = 11)	38.6% (*n* = 26)	*p* < 0.001
Imipenem	5.7% (*n* = 4)	16.7% (*n* = 11)	*p* = 0.041	8.2% (*n* = 6)	24.7% (*n* = 16)	*p* = 0.019
Meropenem	5.7% (*n* = 4)	22.2% (*n* = 15)	*p* = 0.046	6.8% (*n* = 5)	20.8% (*n* = 14)	*p* = 0.028
SMX/TMP ^b^	11.4% (*n* = 10)	46.3% (*n* = 31)	*p* < 0.001	27.4% (*n* = 20)	23.4% (*n* = 15)	n.s.
Colistin	0% (*n* = 3)	0% (*n* = 2)	-	0% (*n* = 8)	0% (*n* = 11)	-

^a^ Comparison of resistance levels among isolates originating from outpatients and inpatients; ^b^ sulfamethoxazole/trimethoprim; Statistical analyses were performed using the Student’s t-test; *p* values < 0.05 were considered statistically significant, n.s.: Not significant.

**Table 2:** Percentage of resistant *Pseudomonas* strains to relevant antibiotics from inpatient and outpatient departments (2008–2017).

Tested Antibiotics	2008–2012			2013–2017		
	Outpatients	Inpatients	Statistics ^a^	Outpatients	Inpatients	Statistics ^a^
Amikacin	18.3% (*n* = 52)	22.1% (*n* = 116)	n.s.	13.1% (*n* = 38)	13.1% (*n* = 69)	n.s.
Gentamicin	31.1% (*n* = 89)	47.4% (*n* = 247)	*p* = 0.043	13.1% (*n* = 38)	25.9% (*n* = 135)	*p* = 0.043
Tobramycin	28.6% (*n* = 82)	44.2% (*n* = 231)	*p* = 0.038	18.2% (*n* = 52)	22.7% (*n* = 119)	n.s.
Ciprofloxacin	34.5% (*n* = 99)	51.2% (*n* = 268)	*p* = 0.033	31.6% (*n* = 91)	38.2% (*n* = 200)	n.s.
Levofloxacin	39.4% (*n* = 113)	54.8% (*n* = 286)	*p* = 0.033	33.9% (*n* = 98)	41.5% (*n* = 217)	n.s.
Imipenem	10.9% *(n* = 31)	22.8% (*n* = 119)	*p* = 0.042	16.2% (*n* = 47)	28.3% (*n* = 148)	*p* = 0.036
Meropenem	12.7% (*n* = 36)	24.7% (*n* = 129)	*p* = 0.04	11.9% (*n* = 34)	26.3% (*n* = 138)	*p* = 0.029
Ceftazidime	9.6% (*n* = 29)	23.1% (*n* = 121)	*p* = 0.036	13.0% (*n* = 37)	15.1% (*n* = 79)	n.s.
Cefepime	14.9% (*n* = 43)	23.3% (*n* = 122)	*p* = 0.045	9.5% (*n* = 27)	12.1% (*n* = 63)	n.s.
Piperacillin/tazobactam	11.2% (*n* = 32)	21.9% (*n* = 115)	*p* = 0.045	16.9% (*n* = 48)	18.4% (*n* = 96)	n.s.
Colistin	0% (*n* = 2)	0% (*n* = 3)	-	0% (*n* = 10)	0% (*n* = 12)	-

^a^ Comparison of resistance levels among isolates originating from outpatients and inpatients; Statistical analyses were performed using the Student’s t-test; *p* values < 0.05 were considered statistically significant, n.s.: Not significant.

## Abbreviations

AST: Antimicrobial susceptibility testing; CES: *Citrobacter-Enterobacter-Serratia*; EUCAST: European Committee on Antimicrobial Susceptibility Testing; ID: Identification; LIS: Laboratory information system; LPS: Lipopolysaccharide; MHA: Mueller-Hinton agar; NFGNB: Non-fermenting Gram-negative bacteria; MALDI-TOF MS: Matrix-assisted laser desorption/ionization time-of-flight mass spectrometry; MDR: Multidrug resistant; SMX/TMP: Sulfamethoxazole/trimethoprim; SPACE: *Serratia-Pseudomonas-Acinetobacter-Citrobacter-Enterobacter*; US: United States; UTI: Urinary tract infections; XDR: Extensively drug resistant.
